# Improvement of Antioxidant Properties in Fruit from Two Blood and Blond Orange Cultivars by Postharvest Storage at Low Temperature

**DOI:** 10.3390/antiox11030547

**Published:** 2022-03-14

**Authors:** Lourdes Carmona, Maria Sulli, Gianfranco Diretto, Berta Alquézar, Mónica Alves, Leandro Peña

**Affiliations:** 1 Instituto de Biología Molecular y Celular de Plantas, Consejo Superior de Investigaciones Científicas, Universidad Politécnica de Valencia, CP 46022 Valencia, Spain; lcarmona@ibmcp.upv.es (L.C.); beralgar@ibmcp.upv.es (B.A.); 2 Fundo de Defesa da Citricultura (Fundecitrus), Sao Paulo 14807-040, Brazil; monicanelialves@gmail.com; 3 Agenzia Nazionale per le Nuove Tecnologie, l’Energia e lo Sviluppo Economico Sostenibile, Centro Ricerche Casaccia, Via Anguillarese, 301, Santa Maria di Galeria, 00123 Rome, Italy; maria.sulli@enea.it (M.S.); gianfranco.diretto@enea.it (G.D.); 4 Faculdade de Ciências Agrárias e Veterinárias (FCAV), Universidade Estadual Paulista (UNESP), Jaboticabal 14884-900, Brazil

**Keywords:** antioxidants, blood oranges, flavonoids, anthocyanins

## Abstract

Numerous studies have revealed the remarkable health-promoting activities of citrus fruits, all of them related to the accumulation of bioactive compounds, including vitamins and phytonutrients. Anthocyanins are characteristic flavonoids present in blood orange, which require low-temperature for their production. Storage at low-temperature of blood oranges has been proven to be a feasible postharvest strategy to increase anthocyanins in those countries with warm climates. To our knowledge, no studies comparing the effect of postharvest storage effect on phenylpropanoid accumulation in cultivars with and without anthocyanins production have been published. We have investigated the effect of postharvest cold storage in flavonoid accumulation in juice from *Citrus sinensis* L. Osbeck in two different oranges: Pera, a blond cultivar, and Moro, a blood one. Our findings indicate a different response to low-temperature of fruit from both cultivars at biochemical and molecular levels. Little changes were observed in Pera before and after storage, while a higher production of phenylpropanoids (3.3-fold higher) and flavonoids (1.4-fold higher), including a rise in anthocyanins from 1.3 ± 0.7 mg/L to 60.0 ± 9.4 mg/L was observed in Moro concurrent with an upregulation of the biosynthetic genes across the biosynthetic pathway. We show that postharvest storage enhances not only anthocyanins but also other flavonoids accumulation in blood oranges (but not in blond ones), further stimulating the interest in blood orange types in antioxidant-rich diets.

## 1. Introduction

**Figure 1:**
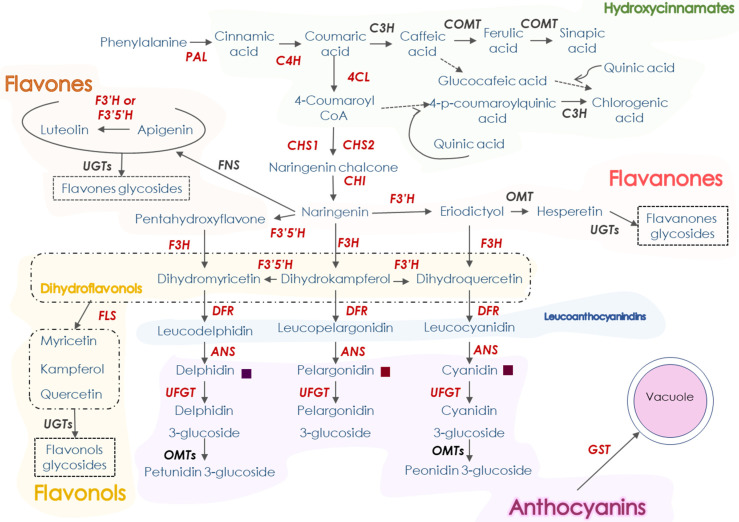
A schematic representation of the phenylpropanoid pathway. Red-bold labelled genes are those studied in this work. Gene names are abbreviated as follows: *PAL*, *phenylalanine ammonia-lyase*; *C3H*, *p-coumarate 3-hydroxylase*, *C4H*, *cinnamate 4-hydroxylase*; *4CL*, *4-hydroxy-cynnamoyl CoA ligase*; *CHS*, *chalcone synthase*; *CHI*, *chalcone isomerase*; *COMT*, *caffeic acid 3-O-methyltransferase*; *FNS*, *flavone synthase*; *F3H*, *flavanone 3-hydroxylase*; *F3′H*, *flavonoid 3′-hydroxylase*; *F3′5′H*, *flavonoid 3′5′-hydroxylase*; *GST, glutathione-S- transferase; OMTs*, *O-methyltransferases*; *FLS*, *flavonol synthase*; *DFR*, *dihydroflavonol 4- reductase*; *ANS*, *anthocyanidin synthase*; *UFGT*, uridine *diphosphate-glucose:flavonoid 3-O-glucosyltransferase* and *UGTs*, *O-methyltransferase*.

Nutraceuticals are phytochemical compounds found in vegetables and fruits, which are getting high consideration for their health-promoting effects when consumed with a certain frequency. Both fruits and vegetables are rich sources of polyphenols, including flavonoids, involved in reducing inflammation and oxidative stress related with chronic diseases, such as those derived from cardiovascular risks, and different types of cancer or diabetes [1,2,3]. Flavonoids, biosynthesised from the phenylpropanoid pathway (Figure 1), constitute the largest class of nutraceuticals in our diet [4]. Depending on their structure, flavonoids can be grouped into six main categories: flavones, flavonols, isoflavones, flavanones, flavanols, and anthocyanins. Among them, flavanones and anthocyanins present a higher antioxidant activity [5].

Citrus fruits are excellent sources of nutrients due to their abundance in vitamin C, sugars, dietary fibre, minerals and phytochemicals, including flavonoids [6,7,8,9,10,11,12]. The vital bioactivities of these secondary metabolites have made blood oranges (BO) to be used as traditional medicine in different Asian countries [13,14]. Flavones are found mainly in their peels, while flavanones are present in both peel and juice of oranges, mandarins, lemons and grapefruits [15]. Regarding anthocyanins, they are only accumulated in peel and juice of the BO, providing not only vivid colours but also a higher antioxidant activity to their pulp and juices [16,17,18]. In fact, it has been proposed that dietary anthocyanins are more effective antioxidants than vitamins E and C [19]. Other beneficial effects of anthocyanins include their anticancer activities, antiviral properties and protective effects against various metabolic, degenerative and cardiovascular diseases as well as eyesight or inhibiting viral replication [20,21,22].

Anthocyanin production in BO is very dependent on cold-temperature, making their quality at harvest to fluctuate between geographical locations and seasons. In specific cold regions of China, Spain and Italy, BO develop optimal colour, while countries with tropical climates such as Sao Paulo (Brazil) or Florida (USA) yield BO with a very low pigmentation [23]. The number of hours of exposition to low-temperature has been proposed as a crucial factor to get a strong purple/red coloration in the fruit [24]. Storage at either 4 or 8 °C has been reported to promote BO characteristic coloration because of the activation of anthocyanin biosynthetic genes [25,26,27,28,29], and by the increase in proteins related to anthocyanin biosynthesis, energy input, and other metabolic pathways associated with defence, oxidative and stress responses [30]. Storage at 9 °C in comparison to 4 °C has been demonstrated to be more effective for enrichment of anthocyanin production in BO [31] and an additional stress treatment (curing) further promotes anthocyanin production, increasing additionally flavonoids accumulation [32]. Low temperature conservation offers also the advantage of being the most frequently used technique to extend the postharvest life and preserve the quality of citrus fruits [33]. Additionally, in order to control pests, several countries require storage at low temperature for citrus fruits exportation to other markets [34].

The consumption of BO may be an important contribution to healthy diets [18,22]. Thus, juices and beverages flaunting red/purple orange in their composition increase their market value, owing to the established health-promoting potential of purple/red orange bioactive components [35]. Besides, increasing flavonoids accumulation in blond oranges by postharvest treatment can also improve the health-promoting properties of derived products or even of fruit consumption. To our knowledge, there is little information comparing the postharvest storage effect on phenylpropanoids accumulation in blond and BO. In this work, we investigated low-temperature storage effect on flavonoids and other phenylpropanoids in two selected cultivars of *Citrus sinensis* (L.) Osbeck, Pera (blond) and Moro (blood). Pera blond sweet orange is the most important citrus cultivar in Brazil (2nd worldwide citrus producer), constituting more than a third of the commercial acreage in São Paulo State [36]. Regarding BOs, Moro, characterised by yielding deeply purple-coloured fruits [17], is the most widely grown cultivar in Europe for food processing and other industrial applications, and the most common BO cultivar grown in the United States [36].

## 2. Materials and Methods

### 2.1. Plant Materials and Storage Conditions

The effect of postharvest storage on fruit colour was determined in two different cultivars of *Citrus sinensis* L. Osbeck, Moro (blood type) and Pera (blond type) mature fruits (8 months after the bloom). Fruits were harvested from adult trees grown (13 years old) and grafted on citrumelo Swingle under standard cultivation in a commercial orchard in Maringá (21°45′53″ S; 48°28′21.15″ O, 540 m), Gavião Peixoto-Sao Paulo State (Brazil). The local climate is Cwa type (mountain subtropical), characterised by dry winter (<1230 mm total rainfall of the year) and a normal average air temperature of ≥17 °C and ≥28 °C in the coldest and in the warmest month, respectively [37]. Maringá soil type is classified as *latossolo vermelho* (Red-oxisol), dystrophic A moderate type, and soft-moderately wavy relief [38]. A total of 80 fruits were harvest from 15 trees planted at 7.0 m × 3.0 m spacing. Fruits were uniform in size and colour, as well as free of damage or external defects. Harvested fruits were stored for 45 days at 9 °C, and 90–95% relative humidity in constant darkness. At the time of harvest (zero) and after 15, 30 and 45 days of storage, pulp and juice samples were taken and stored at −80 °C until analysis. Three replicate samples of 5 fruits each per storage time were used. Pulp was separated with a scalpel, and immediately frozen in liquid nitrogen and ground into a fine powder. Juice was extracted with a domestic squeezer (Braun GmbH, Germany), filtered through a metal sieve with a pore size of 0.8 mm and frozen in liquid nitrogen.

### 2.2. Determination of Internal Maturity Index, pH and Total Anthocyanin Quantification

Titration with phenolphthalein was used to determine the juice acidity, and data were expressed as mg citric acid per 100 mL [31]. Briefly, 5 mL of centrifuged juice were diluted to 45 mL with water (Sigma) and supplemented with 5 drops of phenolphthalein. Acidity was evaluated by titration with NaOH (0.1 N) and expressed as mg of citric acid per 100 mL. The determination of soluble solids content (°Brix) was estimated by refractometer, using an Atago^®^ refractometer (Tokyo, Japan) as described by Carmona et al. [31]. Maturity index was calculated and expressed as the ratio of °Brix/acidity. The pH value was measured with a pH-meter Gehaka^®^ (Sao Paulo, Brasil) [31].

### 2.3. Flavonoids, Anthocyanins and Phenylpropanoid Extraction and Identification

All compounds were extracted using 2 mg of Moro (blood type) or Pera (blond type) mature freeze-dried fruits pulp re-suspended in 20 mL of water (LC-MS Grade, LiChrosolv^®^, Merck, Darmstadt, Germany). Flavonoids and other phenylpropanoids were extracted as previously described by Carmona et al. [32], with the following modifications: 0.5 mL of each re-suspended pulp solution was shaken for 1 h at 20 Hz using a Mixer Mill 300 (Qiagen, Hilden, Germany) with 1.5 mL of methanol containing 0.3% formic acid, plus 2 μg/L of formononetin as the internal standard, and centrifuged at 15,000× *g* for 20 min (15 °C). The supernatants (0.4 mL) were filtered into vials for LC/MS analysis (Mini-UniPrep^®^ syringeless filters with 0.2 μm pore size PTFE membrane, Whatman^®^, Maidstone, UK). LC/MS analysis was performed using an HPLC system equipped with a photodiode array detector (Dionex, ThermoFisher Scientific, Sunnyvale, CA, USA) coupled to a quadrupole-Orbitrap Q-exactive system (ThermoFisher Scientific, Sunnyvale, CA, USA). HPLC analysis was performed using a C18 Luna Column (Phenomenex, Aschaffenburg, Germany) (150 × 2.0 mm; 3 μm). Total of 5 μL of each extract were injected at a flow of 0.25 mL/min. Total run time was 32 min using an elution system running at 0.250 mL/min and consisting of (A) water (0.1% formic acid) and (B) acetonitrile: H_2_O 90:10 (0.1% formic acid). Gradient was 0 to 0.5 min 95/5%-A/B, 24 min 25/75%-A/B, and 26 min 5/95%-A/B. MS analysis of flavonoids and other phenylpropanoids was carried out with a heated electrospray ionization (HESI) source operating in positive and negative ion mode. Mass spectrometer parameters were as follows: sheath and aux gas flow rate set at 40 and 10 units, respectively; capillary temperature was at 250 °C, discharge current was set at 3.5 μA and S-lens RF level at 50. The acquisition was carried out with *m*/*z* 110–1600 Full MS scan range, and the following parameters: resolution 70,000, microscan 1, AGC target 1e6, and maximum injection time 50. Data were analysed using the Xcalibur 4.4 software (ThermoFisher Scientific, Sunnyvale, CA, USA). Metabolites were identified as M+H and M-H adducts, based on their accurate masses (*m*/*z*) and MS fragmentation, using both in house database and public sources (e.g., KEGG, ChemSpider, PubChem, MetaCyc, Metlin, Phenol-Explorer). Relative abundances of the investigated flavonoids and other phenylpropanoids were calculated as fold average and the standard deviation of integrated areas under the *m/z* peak of the adduct of each compound and the internal standard peak area (Fold/ISTD), calculated with the Xcalibur 4.4 software (ThermoFisher Scientific, Sunnyvale, CA, USA).

Anthocyanins were analysed in samples obtained by mixing 500 μL of each re-suspended pulp solution with 500 μL 85:15 methanol:HCl (1 N) containing 0.3% formic acid and 2 μg/L of formononetin (Sigma-Aldrich, San Luis, MO, USA) as internal standard [39]. Samples were shaken for 12 h and subsequently centrifuged for 10 min at 15,300× *g* at 25 °C. Supernatants (0.25 mL) were transferred to new Eppendorf vials, dried by Speedvac concentrator, resuspended in 0.1 mL 75% methanol (plus 0.1% formic acid) and centrifuged (10 min at 15,300× *g* at 25 °C). Total of 70 μL of supernatant was transferred to HPLC vials for LC/MS analysis, and 10 μL of extract was injected to the HPLC-PDA/MS. The method used for separation performed with a C18 Luna column (Phenomenex, Aschaffenburg, Germany) (150 × 2.0 mm; 3 μm) was as previously described [39] and PDA detection was performed by an online Accela Surveyor photodiode array detector (PDA; ThermoFisher Scientific, Sunnyvale, CA, USA), acquiring continuously from 200 to 600 nm. Mass spectrometry analysis was performed using a quadrupole-Orbitrap Q-exactive system (ThermoFisher scientific, Sunnyvale, CA, USA) and ionization was carried out with a heated electrospray ionization (HESI) source operating in positive ion mode. The MS parameters used are as follows: nitrogen was used as sheath and auxiliary gas (45 and 15 units, respectively), capillary and vaporizer temperatures 30 °C and 270 °C, respectively, discharge current 4.0 KV, probe heater temperature at 370 °C, S-lens RF level at 50 V. The acquisition was carried out in the 110–1600 *m/z* scan range, resolution 70,000, microscan 1, AGC target 1e6, and maximum injection time 50 [39]. In detail, MS analysis was performed using a first full scan with data-dependent MS/MS fragmentation in order to identify the anthocyanins in pulp extracts. Subsequently, a single ion monitoring (SIM) with targeted MS/MS fragmentation was applied to identify anthocyanins for which dd-MS/MS fragmentation was not successful, and to further validate the tentative identifications. Anthocyanins were analysed using Xcalibur 3.1 software (ThermoFisher Scientific, Sunnyvale, CA, USA) and identified as M+ adducts, based on their accurate masses (m/z) and MS fragmentation, compared with in house database and public sources (e.g., KEGG, MetaCyc, ChemSpider, PubChem, Metlin, Phenol-Explorer), as well as with comigration with available authentic standards (cyanidin 3-glucoside, peonidin 3-glucoside and dephinidin 3-glucoside) (Extrasynthese, Genay, France). Absolute amounts were measured as previously described [39] using the two most abundant fragments per compound, and data were normalised based on integrated peak areas of external calibration curves of previously described standards [39]. LOD (limit of detection) was estimated from signal-to-noise ratio (S/N) as described [40] and defined as signal intensity corresponding to three times of that noise, while LOQ (limit of quantification) was nine times of that noise. All data are presented as means and standard deviation of at least three independent biological replicates. All the chemicals and solvents used during both the procedures were of LC/MS grade.

### 2.4. Quantitative RT-PCR Analysis

Plant material used for flavonoids and anthocyanins analysis was the same as used for total RNA isolation. Total RNA extraction, DNase treatment, cDNA synthesis and quantitative real-time PCR (qPCR) and relative gene expression were performed as described previously by Carmona et al. [27]. Briefly, qPCR was achieved with a StepOne Plus Real Time PCR System (Applied Biosystem, Waltham, MA, USA) and analysed using StepOne Software version 2.3 (Thermo Fisher, Valence, Spain). RT-PCR was carried out with 50 ng of total cDNA adding 6 µL of SYBR Green PCR Master Mix (Applied Biosystems, Waltham, MA, USA) and 0.3 µM of gene specific primers in a total volume of 12 µL. The RT-PCR procedure consisted of 95 °C 10 min followed by 40 cycles at 95 °C 15 s and 60 °C 40 s. Primers sequences for analysing *phenylalanine ammonia-lyase* (*PAL*), *cinnamate 4-hydroxylase* (*C4H*), *4-hydroxy-cynnamoyl CoA ligase* (*4CL*), *chalcone synthases 1* and *2* (*CHSs*), *chalcone isomerase* (*CHI*), *flavonoid 3-hydroxylase* (*F3H*), *flavonoid 3′5′-hydroxylase* (*F3′5′H*), *flavonol synthase* (*FLS*), *dihydroflavonol 4-reductase* (*DFR*), *anthocyanidin synthase* (*ANS*), *uridine diphos-phate-glucose:flavonoid 3-O-glucosyltransferase* (*UFGT*) and *glutathione-S-transferase* (*GST*) genes are described in Table S1. The relative expression between cold-treated and control samples (zero time of orange fruits) was determined by the method described by Livak et al. [41]. Values are presented as the mean of at least three independent analyses. Statistical analyses were performed using ANOVA.

## 3. Results

### 3.1. Pulp and Juice Appearance and Quality Parameters in Moro and Pera Oranges after Storage

**Figure 2:**
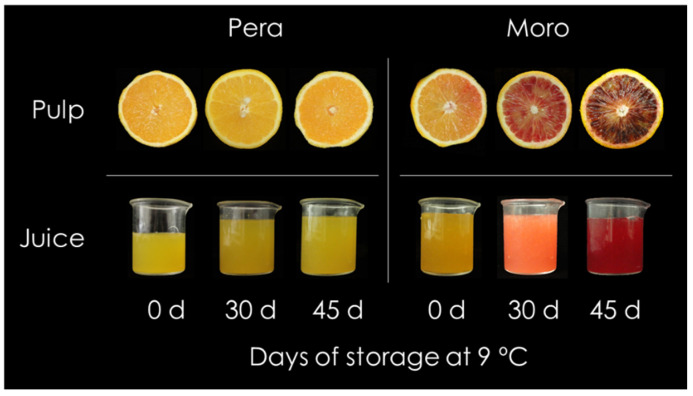
Internal oranges appearance and colour of pulp (**up**) and juices (**down**) of Pera (**left**) and Moro (**right**) oranges from Sao Paulo (Brazil), during storage at 9 °C for 0, 30 and 45 days.

The effect of postharvest storage on visual aspect, maturity index (MI), total flavonoid and anthocyanin contents were assessed in pulp and juice from Moro (blood type) and Pera (blond type) mature oranges subjected to postharvest storage at 9 °C (Figure 2 and Table 1). The visual aspect of pulp and juice was evaluated at the onset, at 30 and 45 days. No changes in colour were detected in pulp and juice from Pera during all the storage period, while a notable enhancement in the red/purple coloration was observed in those from Moro (Figure 2).

No differences were found in MI and pH along the storage period in any of the two fruit types investigated (Table 1). Total flavonoids content was different between both types, being 1.4-fold higher in Moro than in Pera at the onset of the experiment (Table 1). Moreover, no enhanced accumulation was detected in the blond cultivar during the storage period, while an increment of 1.4-fold was observed in Moro at the end of the storage. No anthocyanin presence was detected in Pera orange, while Moro fruit displayed a noticeable presence and considerable enhancement of anthocyanins from 1.3 ± 0.7 mg/L to 60.0 ± 9.4 mg/L under storage conditions.

### 3.2. Accumulation of Hydroxycinnamates and Flavonoids (Non-Anthocyanins) in Moro and Pera Oranges during Postharvest Storage

**Figure 3:**
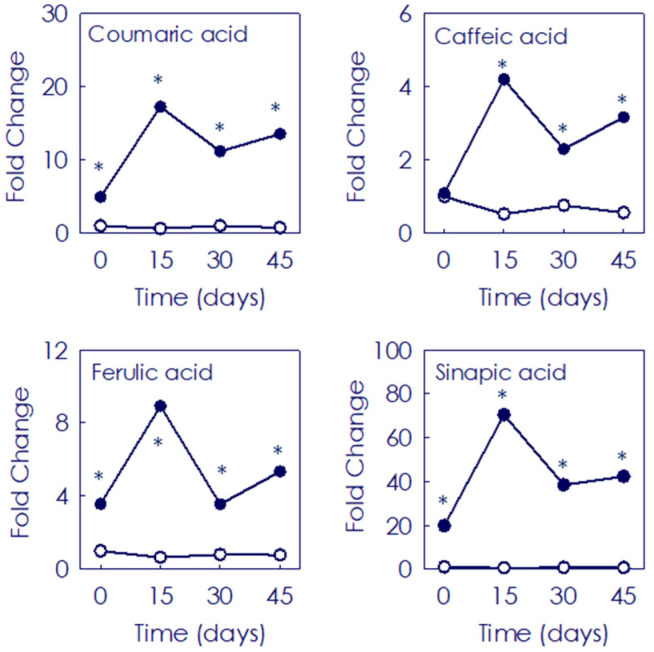
Fold change of the 4 main hydroxycinnamic acids identified in the pulp of Moro (●) and Pera (○) oranges during storage for 0, 15, 30 and 45 days. Data are expressed as the mean fold change ± SD of each sample as compared to the control Pera fruits sample (at harvest time). Asterisk indicates statistically significant different values (*p* ≤ 0.01) for each given time point when comparing cultivars.

Contents of hydroxycinammic acids (HA) and main flavonoids were assessed in the pulp of Pera and Moro fruit during 9 °C post-harvest storage (Figure 3 and Table S2). The profile of the eleven HA identified showed significant differences between both cultivars (Table S2). In general, the BO presented a higher HA content at the onset of the experiment, and a much higher accumulation along all the postharvest experimental period. For instance, the content of coumaric acid, precursor of both HA and flavonoids, was 4.9- and 13.6-fold higher in Moro than in Pera at the onset of the experiment and after 45 days, respectively (Figure 3 and Table S2).

**Figure 4:**
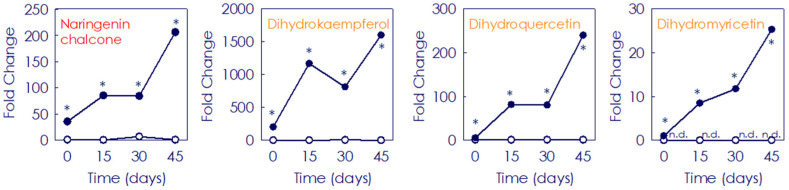
Fold change of naringenin chalcone (flavonoids precursor, in red colour) and the dihydroflavonoids (anthocyanins precursors, in orange colour) dihydrokampherol, dihydroquecetin and dihydromyricetin in the pulp of Moro (●) and Pera (○) oranges during storage for 0, 15, 30 and 45 days. Data are expressed as the mean fold change ± SD of each sample compared to the control Pera fruits sample (at harvest time). Asterisk indicates statistically significant different values (*p* ≤ 0.01) for each given time.

**Figure 5:**
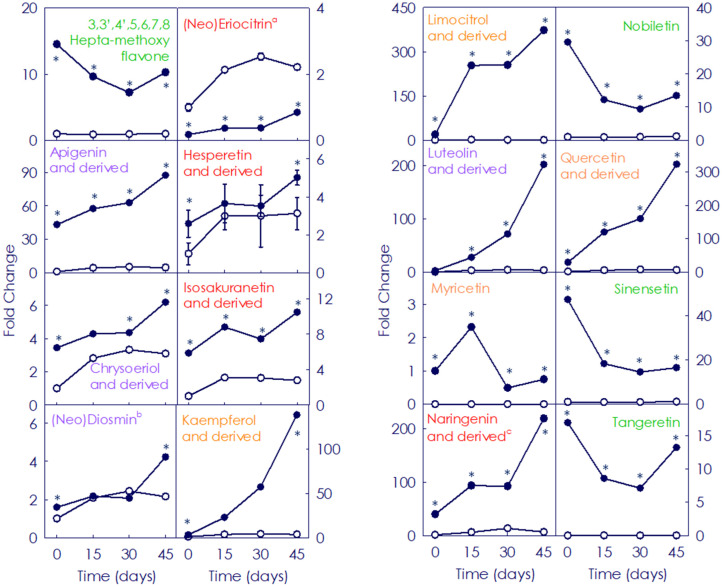
Fold change of representative flavonoids identified in the pulp of Moro (●) and Pera (○) oranges during storage for 0, 15, 30 and 45 days. Compounds belonging to flavanones class are represented in red colour, flavonols class in orange and the flavones in purple and light green (polymethoxyflavones subgroup). ^a^. Sum of eriocitrin and neoeriocitrin. ^b^. Sum of diosmin and neodiosmin. ^c^. Including the naringenin chalcone. Data are expressed as the mean fold change ± SD of each sample compared to the control Pera fruits sample (at harvest time). Asterisk indicates statistically significant different values (*p* ≤ 0.01) for each given time.

A total of 76 flavonoids, including flavanones, flavonols and flavones were identified and measured during the storage period in juice from both fruit types (Figure 4 and Table S2). Among them, the flavonol class was the main group constituting 38.2% of the total, followed by flavones (35.5%) and flavanones (26.3%). Additionally, polymethoxylated derivatives of each class were identified, with polimethoxyflavones (PMFs) being the most represented. The initial flavonoids profile, their content and the accumulation patterns along storage displayed drastic differences between Moro and Pera. Eight flavonoid compounds present in Moro were not detected in Pera fruit either at the onset or after postharvest storage, such as isosakuranetin or the flavonols kaemferol and myricetin, while only two flavonoids (poncirin and natsudaidain) were not present at the beginning in Moro, but were found later and progressively increased with the storage (0.19 and 122.2-fold, respectively) (Table S2). Regarding the initial content of the individual flavonoids, 25 flavonoids showed low (<0.6-fold) accumulation in Moro than in Pera, as the flavanones naringenin or eriocitrin (0.60 an 0.12-fold, respectively). Conversely, 24 flavonoid compounds exhibited a higher content in Moro than in Pera at the onset, which varied between the 2.21-fold increase of the chrysoriol-8-C-glucoside (scoparin) and 202.4-fold enrichment of dihydrokaempherol (Table S2). Considering the content after the storage period, most of the identified flavonoids (54%) were more than two-fold enhanced in Moro compared to Pera, being among them the main precursors naringenin chalcone (206.4-fold) and the dihydroflavonoids dihydrokaemferol and dihydroquercetin (1602.8 and 239.8-fold, respectively). However, 19.7% of the identified flavonoids displayed less than 0.70-fold accumulation in Moro along storage, such as eriocitrin (0.43-fold) or methoxykaempferol-3-*O*-neohesperidoside (0.07-fold) (Figure 4 and Table S2). Individual flavonoids followed also a different accumulation profile during the storage at 9 °C depending on the cultivar. Whereas in Pera most of the identified flavonoids barely changed with the storage, the individual profile varied in Moro depending on each metabolite (Figure 5 and Table S2). In general, main citrus flavanones (isosakuranetin derivatives), flavones (apigenin, luteolin and their derivatives), and flavonols (quercetin, kampferol and their derivatives) increased along storage (1.2 up to 361.6-fold) in Moro fruit (Figure 5). Although other compounds such as 3,3′,4′,5,6,7,8-heptamethoxyflavone, nobiletin or sinensetin showed a decreasing profile (1.4, 2.2 and 2.9-fold, respectively), their content was still higher in Moro than in Pera fruit along the storage period (Figure 5 and Table S2).

### 3.3. Accumulation of Anthocyanins in Moro Orange during Postharvest Storage

**Figure 6:**
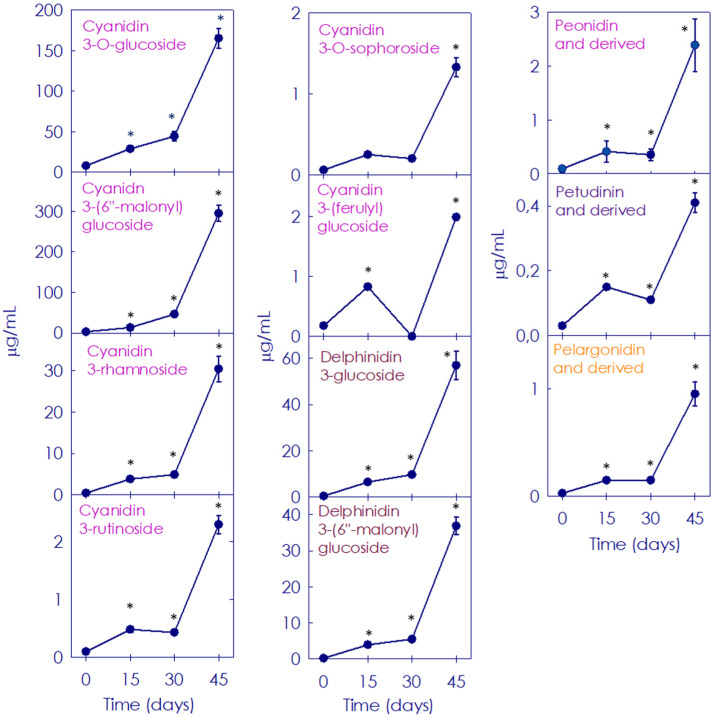
Composition of anthocyanins in the pulp of Moro blood orange during storage for 0, 15, 30 and 45 days. Data are expressed as the mean ± SD of each sample as compared to the control sample (Moro fruit at harvest time). Asterisk indicates statistically significant different values (*p* ≤ 0.01) for each given time.

Variations in anthocyanins composition and contents in Moro orange during post-harvest storage were evaluated for 45 days (Figure 6). No anthocyanins were detected in Pera, while a total of 11 anthocyanins were identified in Moro pulp. At the onset of the study, cyanidin 3-*O*-glucoside (C3-glu) and cyanidin 3-(6″-malonyl)-glucoside (C3-(6M)-glu) were the most abundant anthocyanins in Moro, representing 61.1% and 24.9% of total anthocyanins, respectively. Storage at 9 °C promoted a progressive increase in anthocyanins accumulation, with C3-glu and C3-(6M)-glu remaining as the most abundant ones, representing 27.7% and 49.4% of total anthocyanins at day 45, respectively (Figure 6). Altogether, cyanidin 3-rhamnoside (C3-rha), delphinidin 3-(6″ malonyl)-glucoside (D3-(6M)-glu) and delphinidin 3-glucoside (D3-glu) accounted for 20.8% of total anthocyanins by day 45, in comparison with 9.4% at the onset (Figure 6). At 45 days of storage, the main anthocyanins experimented showed an increase of 5.2-, 4.5-, 3.6- and 3.5-fold for C3-(6M)-glu, C3-rha, Peo3-(6M)-glu and D3-(6M)-glu, respectively. Other minor compounds also increased at the end of the storage period as it was observed for pelargonidin 3-glucoside and cyanidin 3-(ferulyl)glucoside (C3-Fe-glu) (5.3-fold) and cyanidin 3-*O*-sophoride (C3-sph) (2.3-fold). Other anthocyanin pigments that were not found initially, such as petunidin 3,5-glucoside (Pe3,5-glu) and petunidin 3-(6″-malonyl)-glucoside (Pet3-(6M)-glu), were detected at the end of storage (0.14 ± 0.00 and 0.12 ± 0.00 mg/mL, respectively).

### 3.4. Gene Expression Ratio of Phenylpropanoid Biosynthetic Genes between Pera and Moro Orange Pulp during Postharvest Storage

**Figure 7:**
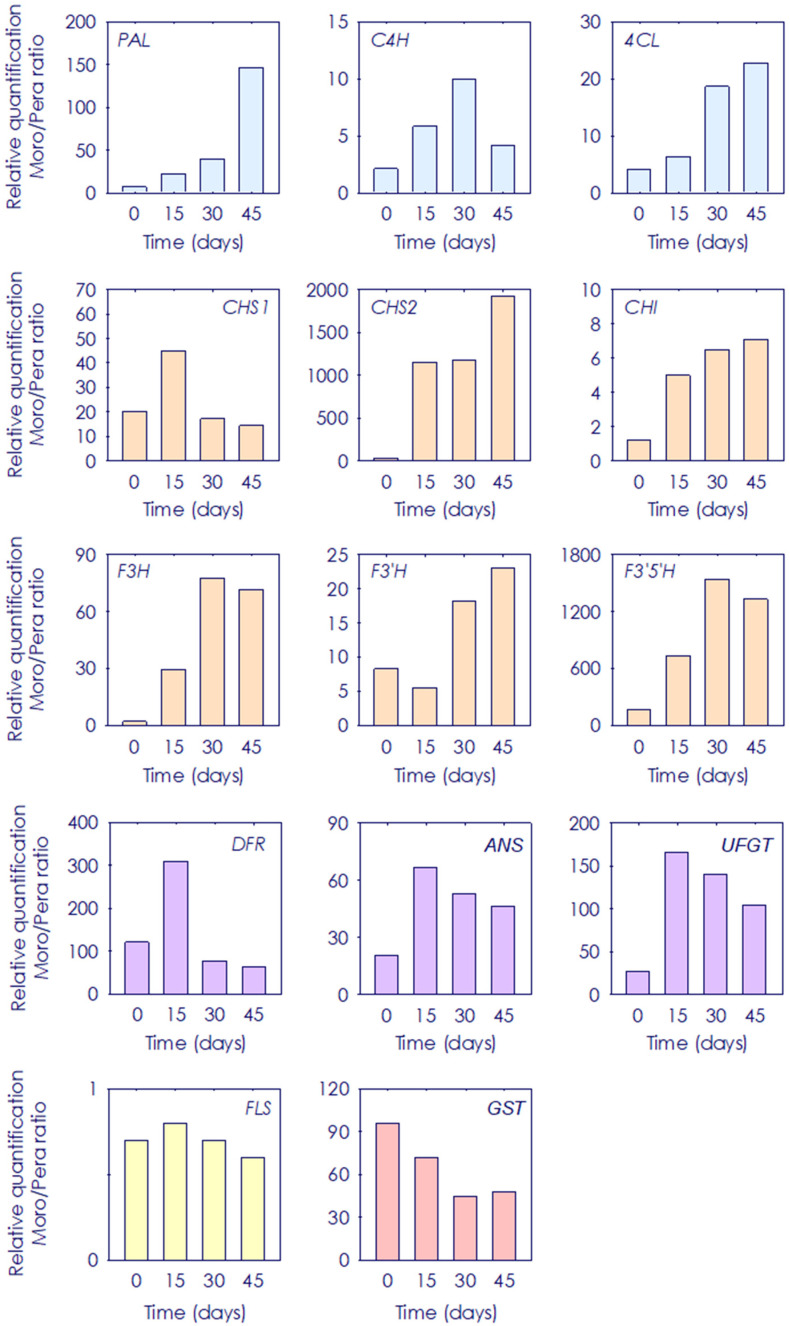
Ratio of the relative expression of *phenylalanine ammonia-lyase* (*PAL*), *cinnamate 4-hydroxylase* (*C4H*), *4-hydroxy-cynnamoyl CoA ligase* (*4CL*), *chalcone synthases 1* and *2* (*CHSs*), *chalcone isomerase* (*CHI*), *flavonoid 3-hydroxylase* (*F3H*), *flavonoid 3’5’-hydroxylase* (*F3’5’H*), *flavonol synthase* (*FLS*), *dihydroflavonol 4-reductase* (*DFR*), *anthocyanidin synthase* (*ANS*), *uridine diphos-phate-glucose:flavonoid 3-O-glucosyltransferase* (*UFGT*) and *glutathione-S-transferase* (*GST*) in the pulp of Moro vs. Pera oranges during storage for 0, 15, 30 and 45 days. Blue, and orange bars indicate genes involved in the general phenypropanoids and flavonoids, respectively. Yellow and purple bars indicate genes involved in the flavonols and anthocyanins biosynthesis, respectively. Red bars belong to those genes involved in the anthocyanin transport.

Transcript accumulation levels between Moro and Pera during postharvest storage were determined and compared as the ratio between relative quantification of Moro vs. Pera at the onset and each postharvest time (Figure 7). A total of 13 genes were evaluated: *PAL*, *C4H* and *4CL* involved in the initial steps of the general phenylpropanoid pathway, seven genes involved in the initial steps of flavonoids biosynthesis (*CHSs*, *CHI*, *F3H*, *F3′H*, *F3′5′H*), three structural anthocyanin biosynthesis genes (*DFR*, *ANS* and *UFGT*) and one gene involved in the transport of the purple/red pigments to vacuoles (*GST*). In general, expression profile revealed that all genes presented a higher ratio at the onset and during the storage period in Moro vs. Pera, with the only exception of *FLS*. At the onset, *F3′5′H* showed up to 168-fold higher expression in Moro than in Pera, and upstream genes *CHS1* and *CHS2* were 20 and 28-fold higher, respectively. Anthocyanin structural gene expression presented also a positive ratio in Moro vs. Pera at the onset of storage, between 20 and 122-fold. During the storage period, the highest induction was shown for *CHS2* and *F3′5′H* with a final ratio of 1915-fold and 1330-fold increase, respectively (Figure 7).

## 4. Discussion

Among fruits, citrus pulp, juice and by-products constitute one of the most important sources of phytonutrients, especially in countries where these fruits are extensively produced. In the last years, the beneficial health-promoting effects claimed for flavonoids has stimulated the interest for investigating these phytochemical compounds in citrus fruits [6,42,43,44,45,46]. Postharvest storage of the fruit at low temperature, combined or not with a stress (i.e., curing) treatment, has been proven to induce anthocyanins and other flavonoids accumulation in BO via stimulation of their biosynthesis [26,28,31]. However, to our knowledge, information regarding changes in the accumulation of specific flavonoid contents in blond and BO under postharvest storage conditions is limited. In this work, the effect of storage on flavonoids and other phenylpropanoids accumulation was investigated and compared in fruits from two important orange cultivars for juice production, Pera (blond) and Moro (blood).

Different responses to the storage at low-temperature were noticed in Pera and Moro fruits. While no visual changes were observed in the blond fruit pulp and juice, Moro fruit displayed a conspicuous enhancement of its purple/red coloration in the pulp and juice, as expected (Figure 2) [31,32]. Juice pH, which can influence anthocyanin by changing their coloration [47], did not vary along the storage period in our study (Table 1). Instead, the progressive darkening of BO pulp and juice could be associated with the increase of anthocyanins content along storage (Figure 2 and Table 1) [31]: from a low content (1.3 ± 0.7 mg/L) at harvest time, it increased 46-fold along storage period due to the cold induction effect (Table 1) [25,26,31,32]. Red/purple colour enhancement in BO during cold storage is related to the greater increase of all individual anthocyanins (Figure 6 and Table 1) [26,28,31,32]. C3-glu and C3-(6M)-glu have been described as the main anthocyanins in BO juice, together with D3-glu, Peo3-(6M)-glu and cyanidin 3-(6″ dioxalyl)-glucoside (C6D-glu) [48]. Accordingly, the two main anthocyanins found in Moro orange were also C3-glu and C3-(6M)-glu, followed by D3-glu and D3-(6M)-glu (Figure 6). In contrast, although no C6D-glu was detected, other anthocyanins such as C3-rha, C3-Fe-glu and C3-sph were accumulated in response to storage at low-temperature in Moro orange (Figure 6) [32]. Due to their electron-donating properties, anthocyanins are potent antioxidants [49]. In the case of BO, the antioxidant activity of anthocyanins was favourable for human health, with impact on some diseases, derived from their anti-inflammatory, anticancer, and antidiabetic properties, due to the prevention of oxidation and free-radical chain reactions [50]. The absence of anthocyanins accumulation in blond orange fruit has been widely documented [23], and is mainly due to the lack of expression in key positive transcriptional factors required for the production of these compounds. Similarly, anthocyanins were not detected in Pera fruit (Table 1).

The beneficial effect of low temperature storage on anthocyanin enhancement has been shown in fruits from other BO cultivars, and was related to a strong boost in the induction and expression of the initial genes of the phenylpropanoids biosynthetic pathway concomitant with the induction of anthocyanin structural genes, leading also to the higher accumulation of other flavonoids [31,32]. Regarding flavonoids, their content and composition in citrus fruits vary among species, cultivars and fruit organs [13,17,51,52,53]. Moreover, stressful temperatures alter the general phenylpropanoid metabolism in citrus fruits [54,55]. In agreement with that, very different accumulation patterns of the individual flavonoid and HA metabolites were observed in fruit from the blood and blond cultivars, the BO pulp being richer at the onset and during postharvest storage (Figure 2 and Figure 3 and Table S2). A higher abundance of HA has been reported in BO fruit compared to that of blond cultivars, being Moro one of the BO types accumulating higher amounts of total HA [17,25]. Additionally, the evaluation of flavonoid profiles revealed a higher initial content and a progressive increment along the storage of the main flavonoids (non-anthocyanins) and anthocyanins only in BO fruit (Figure 4, Figure 5 and Figure 6 and Table S2). Interestingly, storage promoted the enhanced accumulation of precursor substrates in BO, as an effect of a major request of precursor for flavonols and anthocyanins to respond to temperature stress [25,26,31,32]. Concordantly, storage at low temperature induced an increased accumulation of the flavonoid (naringenin chalcone) and anthocyanin (dihydroflavonols) main precursor substrates in Moro in comparison with Pera fruit, concurrent with a higher induction of early phenylpropanoid genes expression (Figure 7) [28,31,32]. Taken together, these results support the higher substrate availability for downstream production of phenylpropanoids in Moro and a different regulation from the initial steps in Moro vs. Pera oranges (Figure 2, Figure 3 and Figure 4 and Table S2) [32].

Flavonoids are potent inducers of antioxidant defence mechanisms in animal cells, by stimulation of different enzymes activity such as glutathione peroxidase, catalase, and superoxide dismutases, or by inhibition of the accumulation of other enzymes such as xanthine oxidase and also the lipid peroxidation as well as protecting other biomolecules, such as DNA from oxidation [43]. In citrus fruits, flavanones have been described as the predominant class of flavonoids, being flavonols the less representative group [51,56]. In our study, flavonoid profiles revealed that the flavonols and flavones classes were the main ones, being flavanones the third (Table S2). These differences might be determined by the different cultivars used [17,52,53], as is supported by the less flavanones accumulation in fruit from blond cultivars stored at low temperature when compared with that of BO cultivars (Table 1 and Table S2) [25]. In our study, although flavanones class was not so representative in the edible portion of the fruit at the onset, storage promoted the increase in the accumulation of hesperidin, hesperitin, naringenin, narirutin or isosakuranetin (and derivatives) (Figure 5 and Table S2). Flavanones from citrus fruits have been awarded important biological activities, as helping in cardiovascular and cancer risk prevention and avoiding the onset of oxidative stress involved in inflammatory damage due to their antioxidant potential [57,58]. The main studies of citrus flavanones antioxidant and anti-inflammatory properties focused on hesperidin and its aglycones (hesperitin), and narirutin [59,60,61,62,63]. In the case of isosakuranetin anti-oxidative activities, they have been related with potential free radical scavenging mechanisms [64]. Numerous studies describing the effects of naringenin on human health reported increasing antioxidant defences, scavenging reactive oxygen species, antiviral responses or exerting anti-atherogenic and anti-inflammatory effects [65]. Naringenin is predominantly found in the edible citrus part and, although is poorly absorbed by oral ingestion, a positive orange juice prebiotic effect due to its bioavailability has been shown [66]. In relation to flavones, compounds grouped into this class also showed a better enrichment in Moro than in Pera fruit under postharvest storage. Among them, luteolin, apigenin and nobiletin (including some glycosylated forms) were the most enhanced by 9 °C storage (Table S2). Citrus flavones have been proposed as the most suitable and capable compounds in terms of antioxidant and anti-inflammatory activities due to their substitutions groups [67]. In the case of nobiletin, it has been indicated to prevent obesity, hepatic steatosis, dyslipidemia, and insulin resistance [42]. Luteolin effects on activation of antioxidant enzymes involved in cancer prevention as well as cardio-protective effects have been reported, as well as properties in inhibiting the onset and development of inflammatory diseases as asthma [68,69,70,71]. Multiple other beneficial bioactivities of apigenin have been proposed on different types of cancer or interactions on gut microbiota [72]. Finally, compounds belonging to the flavonols class were the main groups of flavonoids identified and accumulated in Moro fruit stored at low temperature, mainly limocitrol and quercetin (and their derivatives) (Table S2). Recently, the high intake of flavonol in the diet has been associated to a reduction in the risk of developing Alzheimer dementia [73]. Among the flavonols, limocitrol presented the highest increment observed in our study (Table S2). This compound has been reported as one of the main flavonoids in finger citron (*Citrus medica* cv. sarcodactylis) and their strong antioxidation and antiaging activities have been indicated in both in vitro and in vivo studies [74]. Regarding quercetin and its derivatives, the second most induced flavonol by storage at 9 °C, it is considered as one of the most relevant antioxidant metabolites due to its chemical structure. Its involvement in lipid peroxidation prevention and tocopherol regeneration has been described, as well as in ischemia injury reduction by the induction of nitric oxide synthase [43]. Quercetin has anticarcinogenic and anti-inflammatory properties with antioxidant and free radical scavenging effects. Moreover, other antimicrobial, antiviral, and biological effects, which include anti-inflammatory activity has been attributed to it [75,76].

## 5. Conclusions

BO is an excellent source of natural antioxidant and bioactive compounds, promoting the interest of consumers and researchers in the recent years [6,7]. Many in vivo studies associate the beneficial health-effects of BO juice consumption in reduction of inflammatory processes related to its remarkable antioxidant power [18]. Protection against oxidative stress of flavonoids by induction of reactive oxygen and nitrogen species have been described to play a role as markers of different degenerative diseases [14,18,22]. All these protective bioactivities are likely due to the marked presence of phenolic acids, flavonoids and other phytochemicals in BO [35], although the contribution of each phytochemical in such antioxidant properties requires further research. In this study, storage at low temperature induced a great enrichment on anthocyanins and flavonoids accumulation levels in Moro orange, suggesting that all these compounds could be contributing to their higher antioxidant capacity. However, we did not observe a similar effect of cold storage in Pera fruit. Taken together, we show here that through a regular postharvest practise the content of not only anthocyanins, but also specific health-related flavonoids is enhanced in Moro blood orange pulp and juice (but not in the Pera blond orange counterpart), reinforcing the interest of blood orange to improve natural antioxidant diets. This work should further analyse flavonoids composition and content in other blood and blond orange cultivars to assess whether the drastic increases observed in flavonoids accumulation in Moro fruit during cold storage may be extended to blood-orange types or it is independent of anthocyanins production.

## Figures and Tables

**Table 1:** Maturity index, pH, flavonoids and anthocyanin content of Pera and Moro pulp during storage for 0, 30 and 45 days. Statistical analyses were performed using ANOVA and different letters indicate statistically significant different values (*p* ≤ 0.01) for a given time.

	Pera			Moro		
	0 Days	30 Days	45 Days	0 Days	30 Days	45 Days
Maturity index (MI)	7.9 ± 0.7 ^a^	7.9 ± 0.4 ^a^	7.9 ± 0.6 ^a^	9.5 ± 0.7 ^a^	9.2 ± 0.2 ^a^	9.4 ± 0.1 ^a^
pH	3.8 ± 0.1 ^a^	3.8 ± 0.1 ^a^	3.9 ± 0.0 ^a^	3.6 ± 0.2 ^a^	3.8 ± 0.1 ^a^	3.7 ± 0.0 ^a^
Total flavonoids	22.4 ± 1.5 ^a^	22.1 ± 1.5 ^a^	22.4 ± 1.4 ^a^	31.6 ± 3.3 ^b^	28.2 ± 2.8 ^b^	45.4 ± 2.8 ^c^
Total anthocyanin content (mg/L)	-	-	-	1.3 ± 0.1 ^a^	43.4 ± 4.2 ^b^	60.0 ± 3.5 ^c^

## Data Availability

The data are contained within the article and supplementary material.

## Supplementary Materials

The following supporting information can be downloaded at: https://www.mdpi.com/article/10.3390/antiox11030547/s1. Table S1: Primer sequences used for the quantitative RT-PCR analyses. Table S2: Fold changes of flavonoids identified in the pulp of Pera and Moro orange during storage at 9 °C for 0, 15, 30 and 45 days. Data are expressed as the mean fold change ± SD of each sample as compared to the control sample (harvest time of Pera fruit).
