# Supplementary material for: Improvement of Antioxidant Properties in Fruit from Two Blood and Blond Orange Cultivars by Postharvest Storage at Low Temperature
Source: Antioxidants (Basel). 2022 Mar 14;11(3):547. doi: 10.3390/antiox11030547 (PMC8944625; doi:10.3390/antiox11030547)
Supplement: Supplementary file 1 [file antioxidants-11-00547-s001.zip › Table S1.pdf]

Gene	Acc. Nº[a]	Sequence	Sense
PAL	DQ088064	GATTTGAGACATTTGGAGGA<br>ATGGATGAAGCTCTCCACTA	S<br>AS
C4H	AF255013	CGTGGGAAGGCGAAGCT<br>TCACAAGCCCCAAGATTGGT	S<br>AS
4CL	XM_006478851	CCGAAACGCACCTCATCTG<br>TGATGCCGAGATTCGACAAA	S<br>AS
CHS1	AB009350	GCAGCTATTGGTGTGGTTGG<br>GCACAACATAATTACTTCCCC	S<br>AS
CHS2	AB009351	CCATCAGTATGTTTAGTAGTTC<br>GTTGCCCAGGTTTGGTGAC	S<br>AS
CHI	AB011794	AACAAAGTCGCATTTCCTCGGC<br>GCACGGCGTTCTCCTCCAAGTA	S<br>AS
F3H	AB011795	CCCCTAATTTAAGGCTACCC<br>GACATCGTTCACGAAAACATAC	S<br>AS
F3'H	XM_024189673.1	GGGGCCTGCGGATGGTTC<br>CCTC AACTCTAAGCTTGATAAG	S<br>AS
F3'5'H	HQ634392.1	GAATGGGGATTGTGCTTGTTG<br>CGAGGCGAGGCCGAAGC	S<br>AS
FLS	AB011796.1	CCCGAAAAAGAACAGCCAGC<br>CACTCCCGGCTGGCTTC	S<br>AS
DFR	AY519363	GCTGTTCGTGCTACTGTTC<br>GGCTAAATCGGCTTTCCATA	S<br>AS
ANS	AY581048.1	GGGTGACTGCTAAATGTGTT<br>CAAGTCCCCTGTGAAGAATA	S<br>AS
UFGT	AY519364	TCTTCAGCACTCCGCAAT<br>TCCATCGGATACGTCGTAAG	S<br>AS
GST	EF597102.2	GCAGCAAAGTATGCAAACC<br>GTCATTGAAATTGTGTGCTTC	S<br>AS

[a] Accession number.
